# Non-thermal atmospheric pressure plasma-irradiated cysteine protects cardiac ischemia/reperfusion injury by preserving supersulfides

**DOI:** 10.1016/j.redox.2024.103445

**Published:** 2024-11-28

**Authors:** Akiyuki Nishimura, Tomohiro Tanaka, Kakeru Shimoda, Tomoaki Ida, Shota Sasaki, Keitaro Umezawa, Hiromi Imamura, Yasuteru Urano, Fumito Ichinose, Toshiro Kaneko, Takaaki Akaike, Motohiro Nishida

**Affiliations:** a National Institute for Physiological Sciences (NIPS), National Institutes of Natural Sciences, Okazaki, 444-8787, Japan; b Exploratory Research Center on Life and Living Systems (ExCELLS), National Institutes of Natural Sciences, Okazaki, 444-8787, Japan; c SOKENDAI, Department of Physiological Sciences, Okazaki, 444-8787, Japan; d Center for Novel Science Initiatives (CNSI), National Institutes of Natural Sciences, Tokyo, 105-0001, Japan; e Anesthesia Center for Critical Care Research of the Department of Anesthesia, Critical Care and Pain Medicine, Massachusetts General Hospital, Boston, MA, USA; f Harvard Medical School, Boston, MA, USA; g Organization for Research Promotion, Osaka Metropolitan University, Sakai, 599-8531, Japan; h Graduate School of Medicine, Tohoku University, Sendai, 980-8575, Japan; i Graduate School of Engineering, Tohoku University, Sendai, 980-8579, Japan; j Tokyo Metropolitan Institute for Geriatrics and Gerontology, Tokyo, 173-0015, Japan; k Organization of Research Initiatives, Yamaguchi University, Yamaguchi, 753-8515, Japan; l Graduate School of Pharmaceutical Sciences, The University of Tokyo, Tokyo 113-0033, Japan; m Graduate School of Medicine, The University of Tokyo, Tokyo, 113-0033, Japan; n Graduate School of Pharmaceutical Sciences, Kyushu University, Fukuoka, 812-8582, Japan

**Keywords:** Non-thermal plasma, Supersulfides, Mitochondrial energy metabolism, SQOR, Ischemia/reperfusion

## Abstract

Ischemic heart disease is the main global cause of death in the world. Abnormal sulfide catabolism, especially hydrogen sulfide accumulation, impedes mitochondrial respiration and worsens the prognosis after ischemic insults, but the substantial therapeutic strategy has not been established. Non-thermal atmospheric pressure plasma irradiation therapy is attracted attention as it exerts beneficial effects by producing various reactive molecular species. Growing evidence has suggested that supersulfides, formed by catenation of sulfur atoms, contribute to various biological processes involving electron transfer in cells. Here, we report that non-thermal plasma-irradiated cysteine (Cys∗) protects mouse hearts against ischemia/reperfusion (I/R) injury by preventing supersulfide catabolism. Cys∗ has a weak but long-lasting supersulfide activity, and the treatment of rat cardiomyocytes with Cys∗ prevents mitochondrial dysfunction after hypoxic stress. Cys∗ increases sulfide-quinone oxidoreductase (SQOR), and silencing SQOR abolishes Cys∗-induced supersulfide formation and cytoprotection. Local administration of mouse hearts with Cys∗ significantly reduces infarct size with preserving supersulfide levels after I/R. These results suggest that maintaining supersulfide formation through SQOR underlies cardioprotection by Cys∗ against I/R injury.

## Graphical abstract

**Figure:**
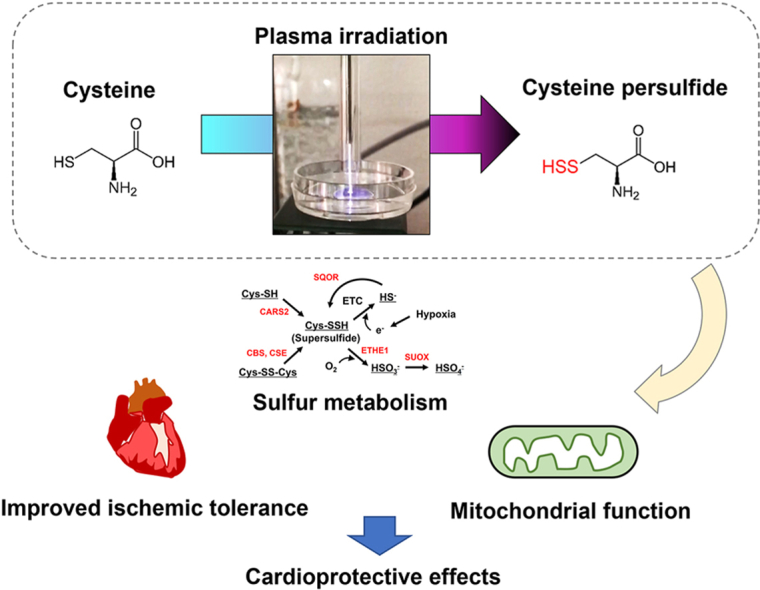

## 1 Introduction

Ischemic heart disease remains the leading cause of cardiovascular-related mortality and chronic heart failure worldwide, despite significant advances in the physician's ability to initiate myocardial reperfusion and salvage heart tissue. Many therapeutic strategies, especially medical interventions to potentiate the heart's resistance to ischemic injury have been intensively investigated, but an efficacious therapy has yet to be successfully implemented in the clinical arena [1]. It remains an urgent issue how to preserve the energy content of cardiomyocytes during ischemia.

In an anaerobic environment, NO_3_^−^, NO_2_^−^, Fe^3+^, SO_4_^2−^, and CO_2_ are used as terminal electron acceptors for microbial respirations [[2], [3], [4], [5], [6]]. In mammalian cells, fumarate reportedly acts as a terminal electron acceptor to sustain a circuit of electron flow in the electron transport chain (ETC) that maintains mitochondrial functions under hypoxia [7]. However, the heart and skeletal muscle have less fumarate reduction activity and will rapidly suppress mitochondrial functions under oxygen limitation. Therefore, to find an oxygen-independent electron transporting mechanism in myocardial mitochondria is expected to establish a new therapeutic strategy for ischemic heart disease. Supersulfides, in which sulfur atom is catenated, have recently attracted attention as true biomolecules with high electron transfer (i.e., redox) reactivity to support energy metabolism and signal transduction [8,9]. Supersulfides emerge both electrophilic and nucleophilic properties, and form hydropolysulfides (R–S(S)_n_H; n ≥ 1 and R ≠ H) and polysulfides (R–S(S)_n_S-R) in cells. In particular, cysteine persulfide (CysSSH) and glutathione persulfide (GSSH) have been found to exist in the micromolar range within the organism and act as antioxidants through their potent nucleophilicity [10]. Increased level of persulfides is implicated in lifespan extension across species [11,12]. We have previously reported that protein-SSH is mainly formed by mitochondria-localized cysteinyl-tRNA synthetase (CARS) 2 by dual functions: CysSSH synthase and cysteinyl-tRNA synthetase activities [8]. CysSSH is also produced by cystathionine-β-synthase (CBS) and Cystathionine-γ-lyase (CSE) using cystine as a substrate [10]. In contrast, supersulfides are catabolized to form H_2_S at mitochondrial ETC [8,13,14] and H_2_S overproduction leads to mitochondrial dysfunction by inhibiting complex IV [15]. We have also reported that supersulfide catabolism is enhanced in failing cardiomyocytes and reduced supersulfides disrupt mitochondrial quality and function by decreasing protein polysulfidation, suggesting the critical role of supersulfides in maintaining cardiac homeostasis and robustness [[16], [17], [18]].

Non-thermal plasma at atmospheric pressure has been successfully employed for potential therapeutic purposes, such as selective elimination of cancer cells [19], promotion of wound healing [20], and gene transfection [21]. Plasma irradiation is accompanied by dissociation/reconstruction of molecular bonds, in a way that is distinct from ion interactions in liquids or the behavior of shared ions in metals. Thus, non-thermal plasma irradiation is expected to produce unique reactive species with unprecedented biochemical properties [20]. Until recently, since hydrogen peroxide is abundantly produced in non-thermal plasma-irradiated culture media, the biological effect of non-thermal plasma irradiation has been widely regarded as the inducer of oxidative stress [22]. However, it has been demonstrated that non-thermal plasma-irradiated solution can also stimulate reactive oxygen species (ROS)-independent signaling pathways to eliminate cancer cells, when starting materials are optimized (i.e. lactate) [23]. Additionally, when HEPES-containing buffer is treated with non-thermal plasma irradiation, short-lived reactive species are estimated to be produced that elicit cytoplasmic Ca^2+^ influx through a ROS-independent mechanism [24,25].

In this study, we report that non-thermal plasma-irradiated cysteine (Cys) solution protects mouse hearts against ischemia/reperfusion (I/R) injury and rat cardiomyocytes against oxygen glucose deprivation/reoxygenation (OGD/R) injury by preserving supersulfide metabolism. Although the chemical supersulfide activity in plasma-irradiated Cys is estimated only ∼80 nM of inorganic sulfane sulfur Na_2_S_4_, the cardioprotective effect of plasma-irradiated Cys can be maintained much longer than that of Na_2_S_4_. We also show that sulfide-quinone oxidoreductase (SQOR) participates in plasma-irradiated Cys-induced cardioprotection against I/R injury by preserving supersulfide metabolism, suggesting a potential therapeutic strategy for ischemic heart disease.

## 2 Results

### 2.1 Plasma-irradiated culture medium containing Cys has a cardioprotective effect

**Fig. 1:**
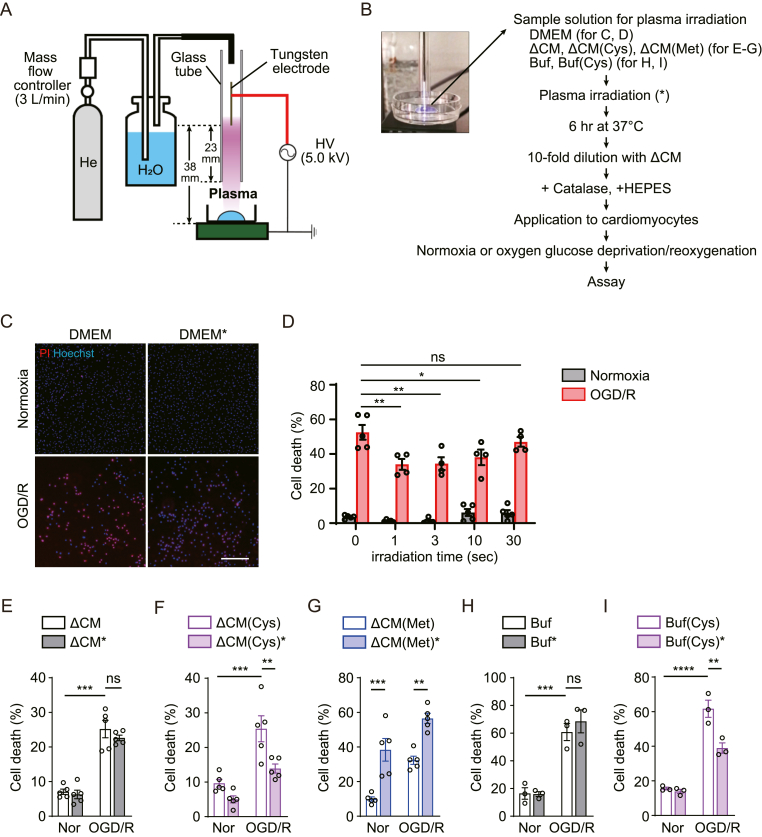
Cardioprotective effect of plasma-irradiated Cys medium (**A**) Experimental setup for the helium atmospheric-pressure plasma (He-APP) irradiation. Inlet: plasma plume reaching the target solution (250 μL) placed on a glass bottom dish. (**B**) Protocol of applying plasma-irradiated solutions to cultured cardiomyocytes. Several types of sample solutions were plasma-irradiated and incubated for 6 h at 37 °C before dilution with methionine and cysteine-free DMEM (ΔCM). Catalase and HEPES were supplemented in all media right before cellular application. ΔCM(Cys), ΔCM supplemented with cysteine; ΔCM(Met), ΔCM supplemented with methionine; Buf, carbonate buffer; Buf (Cys), Buf supplemented with cysteine. (**C**) Effect of plasma-irradiated DMEM (DMEM∗) on cardiomyocyte injury under normoxia or oxygen glucose deprivation/reoxygenation (OGD/R) condition. Cardiomyocytes were stained by PI (red) and Hoechst (blue). Plasma irradiation time, 3 s. Scale bar, 400 μm. (**D**) Cell death rate of cardiomyocytes treated with DMEM∗ with different irradiation times (1–30 s) (n = 4–5 independent experiments). (**E**) Cell death rate of cardiomyocytes treated with plasma-irradiated methionine and cysteine-free DMEM (ΔCM∗) under normoxia (Nor) or OGD/R (n = 5 independent experiments). (**F**, **G**) Cell death rate of cardiomyocytes treated with plasma-irradiated ΔCM supplemented with cysteine or methionine (ΔCM(Cys)∗or ΔCM(Met)∗, respectively) (n = 5 independent experiments). (**H**, **I**) Cell death rate of cardiomyocytes treated with plasma-irradiated carbonate buffer supplemented without or with cysteine (Buf∗ or Buf (Cys)∗, respectively) (n = 3 independent experiments). Data are shown as the means ± SEM. ∗P < 0.05, ∗∗P < 0.01, ∗∗∗P < 0.001 by one-way ANOVA. “ns” indicates not significant. (For interpretation of the references to color in this figure legend, the reader is referred to the Web version of this article.)

Using the setup for non-thermal plasma irradiation using helium as the carrier gas as previously described (Fig. 1A) [26] we first examined whether plasma-irradiated culture medium has a cardioprotective effect. To this end, we treated cardiomyocytes with plasma-irradiated DMEM (DMEM∗), and evaluated its effect on the survival rate following OGD/R stress [27]. After plasma irradiation, a huge variety of short-lived reactive species have been generated, leading to cytotoxicity [28,29]. To eliminate these short-lived reactive species, DMEM∗ was incubated for 6 h at 37 °C. Because the incubated plasma solution still has a high amount of hydrogen peroxide (H_2_O_2_), DMEM∗ was then preincubated with catalase to remove H_2_O_2_ (Fig. 1B). This DMEM∗ containing stable plasma products was used for cardioprotection assay. OGD/R stress increased propidium iodide (PI)-positive cardiomyocyte death, which was inhibited by DMEM∗ treatment (Fig. 1C). When irradiation time is shorter (1 s, 3 s and 10 s), the increase in cell death rate by OGD/R was significantly attenuated by the application of DMEM∗ (Fig. 1D). On the other hand, there was no significant difference between the control group when cells were treated with 30 sec-irradiated DMEM (Fig. 1D). These results suggest that the cardioprotective effect of DMEM∗ is dependent on irradiation time.

We next tested the hypothesis that sulfur-containing components of DMEM acquire cardioprotective effects by plasma irradiation. Because cysteine is a thiol-containing amino acid involved in numerous biological redox reactions [30] and sulfur metabolites have cardioprotective effects [31], we examined whether the effect of DMEM∗ was due to plasma-dependent redox reactions involving Cys. To this end, we used DMEM without cysteine and methionine (ΔCM). No cardioprotective effect was observed in cardiomyocytes treated with plasma irradiated ΔCM (ΔCM∗) (Fig. 1E). Next, ΔCM medium added back Cys or methionine (Met) was plasma-irradiated (ΔCM(Cys)∗ or ΔCM(Met)∗, respectively). While treatment of ΔCM(Cys)∗ significantly attenuated the increase in cell death rate by OGD/R (Fig. 1F), no significant difference was observed in cardiomyocytes treated with non-irradiated ΔCM(Cys) (comparison of Fig. 1E and F). In contrast, cells incubated with ΔCM(Met)∗ exhibited a significant increase in cell death rate regardless of oxygen concentration (Fig. 1G), while no significant difference was observed by ΔCM(Met) treatment (comparison of Fig. 1E and G). We confirmed that the concentration of H_2_O_2_ in ΔCM(Cys)∗ and ΔCM(Met)∗ was below the detection limit after catalase pretreatment (Supplementary Fig. 1). Although these results suggest the necessity of Cys in plasma-irradiated DMEM, DMEM contains various components and it would be difficult to determine whether Cys is a direct target of plasma irradiation. Therefore, we next tested the cardioprotective effect of plasma-irradiated simple Cys buffer. Carbonate buffer without or with Cys was plasma-irradiated (Buf∗ or Buf (Cys)∗, respectively). After 6 h incubation, these solutions were diluted into DMEM and mixed with catalase and HEPES for application to cardiomyocytes. Buf (Cys)∗ but not Buf∗ showed the cardioprotective effect against OGD/R stress (Fig. 1H and I), supporting the involvement of plasma-irradiated Cys products. Taken together, it is suggested that the cardioprotective effect of DMEM∗ is attributed to plasma irradiated Cys products not H_2_O_2_, while cell toxicity of ΔCM(Met)∗ is not due to that of methionine itself. Additionally, Buf (Cys) treatment showed a little higher cytotoxicity compared with ΔCM(Cys) under normoxia (comparison of Fig. 1F and I). Therefore, we mainly use ΔCM(Cys) and ΔCM(Cys)∗ for biological assays.

### 2.2 Supersulfides are formed in plasma-irradiated Cys solution

**Fig. 2:**
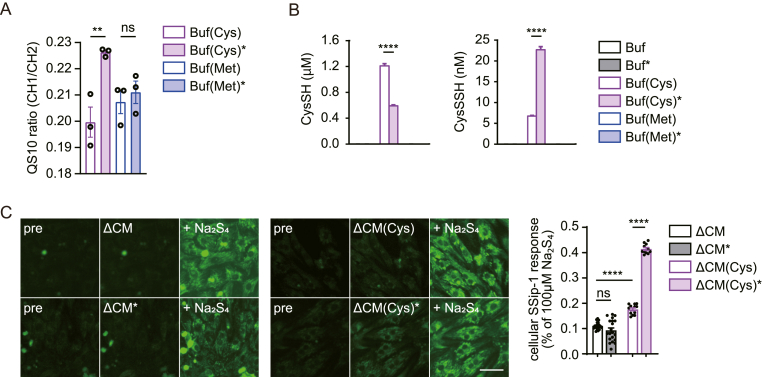
Acute application of plasma-irradiated Cys products increases intracellular supersulfides in cardiomyocytes (**A**) Measurement of QS10 fluorescence ratio (CH1/CH2) in plasma-irradiated cysteine or methionine-containing carbonate buffer (Buf (Cys)∗ or Buf (Met)∗) at 6 h post-irradiation (n = 3 independent experiments). (**B**) Quantitative analysis of HPE-IAM-trapped polysulfides by LC-MS/MS method in plasma-irradiated solutions (n = 3 independent experiments). CysSH, cysteine; CysSSH, cysteine persulfide. (**C**) Intracellular supersulfide imaging. Cardiomyocytes were preloaded with SSip-1 DA, and treated with different plasma-irradiated medium for 15 min. At the end of the experiment, 100 μM Na_2_S_4_ was applied to measure the maximal response. ΔCM∗, plasma-irradiated methionine and cysteine-free DMEM; ΔCM(Cys)∗, plasma-irradiated ΔCM supplemented with cysteine. Scale bar, 50 μm. Bar graph: quantification of SSip-1 intensity normalized by those treated with 100 μM Na_2_S_4_ (n > 11 areas from three independent experiments). Data are shown as the means ± SEM. ∗∗P < 0.01, ∗∗∗∗P < 0.0001 by one-way ANOVA. “ns” indicates not significant.

Supersulfides are endogenously synthesized from sulfide, Cys and cystine (Cys–SS–Cys) [10,17,32] and have a cardioprotective role [17,18,33,34]. We therefore hypothesized that plasma irradiation generates supersulfides from a Cys-containing solution. To test this, we examined the supersulfide level in Buf (Cys)∗ by QS10, fluorescent probes that specifically detect hydropolysulfides (R-(S)_n_SH) [35]. It is important to note that these probes do not respond to H_2_S or sulfide donors (e.g. Na_2_S solution). Only Buf (Cys)∗ exhibited a significantly increased fluorescent signal of QS10 (Fig. 2A). To quantify supersulfide titer of Buf (Cys)∗, the QS10 FRET ratio of the indicated concentrations of Na_2_S_4_ was measured. Supersulfide titer in Buf (Cys)∗ was comparable to about 80 nM Na_2_S_4_ (Supplementary Fig. 2).

Consistently, LC-tandem mass spectrometry (LC-MS/MS) with supersulfide-selective trapping method [36] revealed that Buf (Cys)∗ resulted in ∼2-fold reduction in cysteine (CysSH) and ∼4-fold increase in cysteine persulfide (CysSSH) (Fig. 2B). The levels of oxidized Cys metabolites, such as sulfite (HSO_3_^−^) and thiosulfate (HSSO_3_^−^), were also increased in Buf (Cys)∗ compared to Buf (Cys) (Supplementary Fig. 3). However, no CysSH or its metabolites were detected by LC-MS/MS in Buf∗ and Buf (Met)∗ (Fig. 2B and Supplementary Fig. 3). These results suggest that plasma irradiation generates supersulfides from Cys, but not from Met.

### 2.3 Acute application of plasma-irradiated Cys products increase intracellular supersulfides in cardiomyocytes

Next, we assessed whether ΔCM(Cys)∗ could increase intracellular supersulfide level. To test this, we visualized intracellular supersulfides using fluorescent probe SSip1-DA [37]. ΔCM(Cys)∗ application induced ∼ two-fold increase in intracellular SSip-1 response within 15 min, compared to ΔCM(Cys), ΔCM and ΔCM∗ treatment (Fig. 2C). This result suggests that ΔCM(Cys)∗ may exert cardiomyocyte protective effects via increasing intracellular supersulfide level.

### 2.4 Plasma-irradiated Cys products restore sulfide metabolism under hypoxia via SQOR expression

**Fig. 3:**
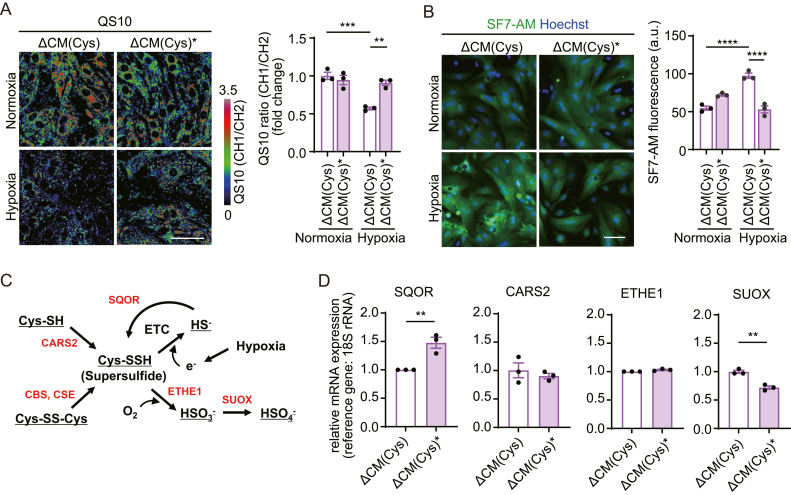
Plasma-irradiated Cys medium improves sulfide metabolism under hypoxia through SQOR expression (**A, B**) Supersulfides and H_2_S imaging in cardiomyocytes. Cardiomyocytes treated with Cys or Cys plasma medium were cultured under normoxia or hypoxia. QS10 (ratiometric, pseudocolored) imaging for supersulfides (**A**), or SF7-AM (green) and Hoechst (blue) imaging for H_2_S (**B**). ΔCM(Cys)∗, plasma-irradiated methionine and cysteine-free DMEM supplemented with cysteine. Scale bars, 50 μm. Bar graph: Quantification of QS10 CH1/CH2 ratio or SF7-AM fluorescence intensity (n = 3 independent experiments). (**C**) Schematic illustration of supersulfide synthesis/catabolism pathway. Related enzymes are shown in red. (**D**) Relative mRNA expression levels of genes involved in supersulfide synthesis in cardiomyocytes (n = 3 independent experiments). Data are shown as the means ± SEM. ∗P < 0.05, ∗∗P < 0.01, ∗∗∗P < 0.001, ∗∗∗∗P < 0.0001 by one-way ANOVA (**A**, **B**); unpaired *t*-test (**D**). (For interpretation of the references to color in this figure legend, the reader is referred to the Web version of this article.)

It has been demonstrated that supersulfides such as CysSSH are catabolized to H_2_S by receiving electrons from the mitochondrial ETC as an electron acceptor [8], and supersulfide conversion to H_2_S is accumulated in hypoxic cardiomyocytes and ischemic heart [17]. We incubated cardiomyocytes in ΔCM(Cys) or ΔCM(Cys)∗ medium under hypoxic conditions and compared the intracellular level of supersulfides and H_2_S under hypoxia for 24 h by fluorescent probe QS10 and SF7-AM, respectively. Long-term treatment of ΔCM(Cys)∗ did not increase the intracellular supersulfide level in normoxic cardiomyocytes, whereas significantly recovered the decreased supersulfides under hypoxia (Fig. 3A). Consistent with this, cardiomyocytes cultured in ΔCM(Cys)∗ exhibited significantly lower signal intensity of SF7-AM compared to Cys-treated cells under hypoxia (Fig. 3B), suggesting improved sulfide metabolism under hypoxia by ΔCM(Cys)∗ treatment. Additionally, Buf (Cys)∗ administration also improved the supersulfide level in hypoxic cardiomyocytes (Supplementary Fig. 4). Individual sulfur resides of supersulfides have both nucleophilic and electrophilic activities [38]. Supersulfides are oxidized to hydrogen sulfate (HSO_4_^−^) by persulfide dioxygenase (ETHE1) and sulfite oxidase (SUOX), whereas are reduced to H_2_S by electrons in ETC [8]. Accumulated H_2_S is oxidized back to persulfides by sulfide-quinone oxidoreductase (SQOR) (Fig. 3C) [14]. We checked the effect of ΔCM(Cys)∗ treatment on the expression level of sulfide metabolism-related genes and found that there was a 1.5-fold increase in Sqor mRNA and 30 % decrease in Suox mRNA in cardiomyocytes cultured in ΔCM(Cys)∗, compared to ΔCM(Cys) under normoxia, suggesting that ΔCM(Cys)∗ maintains intracellular supersulfides via SQOR. Moreover, cardiomyocytes have generally higher antioxidant capacity than other cell types, along with higher expression levels of enzymes involved in sulfide metabolism [14]. Indeed, while sulfide donor Na_2_S treatment induced a rapid increase in intracellular H_2_S levels in HeLa cells, there was no apparent change in cardiomyocytes (Supplementary Fig. 5A). Instead, there was a stark increase in intracellular supersulfides in Na_2_S-treated cardiomyocytes (Supplementary Fig. 5B). Importantly, supersulfide accumulation by Na_2_S was drastically inhibited by the knockdown of SQOR (Supplementary Fig. 5B). These results suggest that SQOR has a critical role in maintaining the proper balance of supersulfides and H_2_S.

### 2.5 Plasma-irradiated Cys products facilitate mitochondrial energy metabolism under hypoxia

**Fig. 4:**
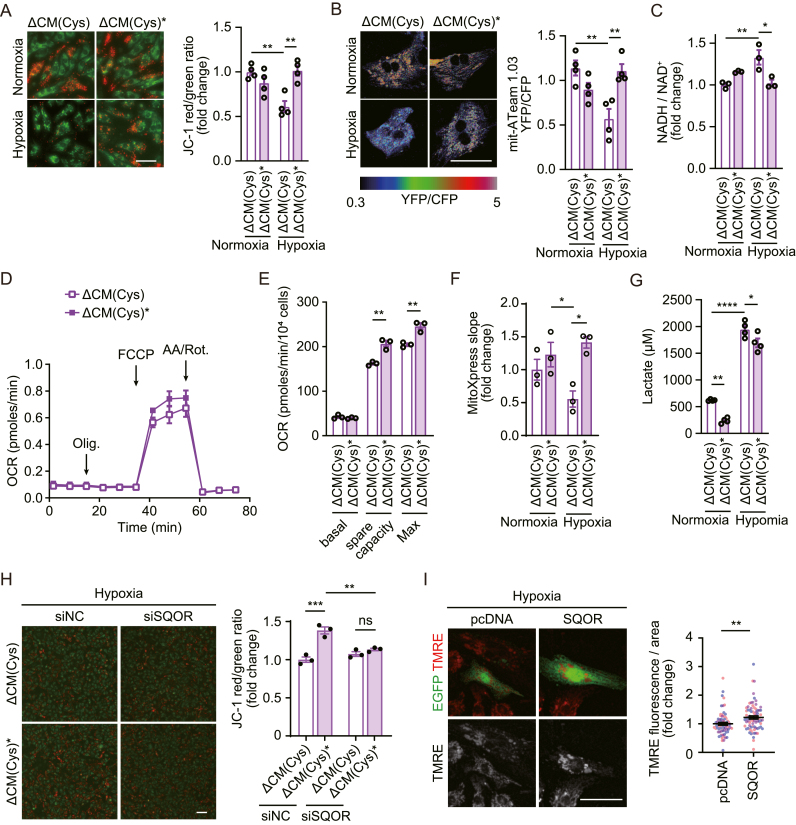
Plasma-irradiated Cys products restore mitochondrial energy metabolism in cardiomyocytes under hypoxia. (**A**) Representative images of cardiomyocytes labeled with JC-1 dye to measure mitochondrial membrane potential. Cardiomyocytes treated with Cys or Cys plasma medium were cultured under normoxia or hypoxia. ΔCM(Cys)∗, plasma-irradiated methionine and cysteine-free DMEM supplemented with cysteine. Scale bars, 50 μm. Right: quantification of JC-1 red/green fluorescence ratio (n = 4 independent experiments). (**B**) Representative YFP/CFP ratiometric pseudocolored images of cardiomyocytes transfected with mit-ATeam 1.03 to monitor mitochondrial ATP. Scale bars, 50 μm. Right: quantification of YFP/CFP FRET ratio (n = 4 independent experiments). (**C**) NADH/NAD^+^ ratio in cardiomyocyte lysates (n = 3 independent experiments). (**D**) Real-time oxygen consumption rate (OCR) measurements of cardiomyocytes cultured in plasma-irradiated Cys medium by Seahorse extracellular flux analyzer under normoxia. Cells were treated with oligomycin (Olig.), FCCP, rotenone (Rot.) and antimycin A (AA). (**E**) Measurements of OCR for different respiration modes (n = 3 independent experiments). (**F**) OCR measurements by MitoXpress Xtra assay. Cardiomyocytes were cultured under Nor or Hyp for 24 h, then basal respiration was measured under Nor (n = 3 independent experiments). (**G**) Lactate concentration in media was measured (n = 4 independent experiments). (**H**) Cardiomyocytes were labeled with JC-1 dye after gene silencing by siRNA transfection under Hyp, and JC-1 red/green fluorescence ratio was quantified. siNC: siRNA for negative control, siSqor: siRNA for Sqor gene (n = 3 independent experiments). Scale bars, 100 μm. (**I**) Cardiomyocytes transfected with EGFP and SQOR or empty vector (pcDNA) were labeled with TMRE to measure mitochondrial membrane potential. Right: Measurement of TMRE fluorescence intensity normalized by EGFP-positive cell surface area (n > 72 cells from 3 independent experiments). Scale bars, 50 μm. Data are shown as the means ± SEM. ∗P < 0.05, ∗∗P < 0.01, ∗∗∗P < 0.001, ∗∗∗∗P < 0.0001 by one-way ANOVA (A-C, F–H); unpaired *t*-test (E, I). “ns” indicates not significant. (For interpretation of the references to color in this figure legend, the reader is referred to the Web version of this article.)

The prolonged hypoxic condition leads to impaired oxidative phosphorylation through the mitochondrial ETC [27], resulting in various pathologies such as myocardial infarction [39]. Because supersulfides such as CysSSH act as an electron acceptor from ETC, thereby facilitating mitochondrial energy metabolism [8,14,40], we investigated whether plasma-irradiated Cys products have a similar effect on mitochondrial function under hypoxia. To test this, we cultured cardiomyocytes in ΔCM(Cys)∗ medium and evaluated the metabolic status using several indicators: mitochondrial membrane potential, level of mitochondrial ATP using FRET-based biosensor mit-ATeam1.03 [27,41], NADH/NAD^+^ ratio and oxygen consumption rate (OCR). Cardiomyocytes under hypoxia exhibited depolarized mitochondrial membrane potential (decreased JC-1 red/green ratio) and decreased ATP production in mitochondria (Fig. 4A and B). Incubation in ΔCM(Cys)∗ medium significantly attenuated the decrease in mitochondrial membrane potential and ATP production in cardiomyocytes under hypoxia, compared to ΔCM(Cys) medium (Fig. 4A and B). Buf (Cys)∗ but not Buf∗ treatment also improved mitochondrial membrane potential under hypoxia (Supplementary Figs. 6A and B). Hypoxia-induced increase in NADH/NAD^+^ ratio was significantly attenuated by ΔCM(Cys)∗ (Fig. 4C). Cardiomyocytes cultured in ΔCM(Cys)∗ had ∼30 % higher spare and maximal respiration rates than cells maintained in ΔCM(Cys) media under normoxia, as analyzed by the Seahorse extracellular flux analyzer (Fig. 4D and E). In addition, cardiomyocytes cultured in ΔCM(Cys) or ΔCM(Cys)∗ under hypoxia are returned to normoxia and OCR of basal respiration was rapidly measured by MitoXpress Xtra assay. ΔCM(Cys)∗ treatment significantly restored the decrease in OCR of basal respiration under hypoxia (Fig. 4F). Impaired ETC/energy production triggers mitochondrial ROS production. Hypoxia-induced mitochondrial ROS production was attenuated by Buf (Cys)∗ but not Buf∗ treatment (Supplementary Fig. 6C). These results demonstrate that plasma-irradiated Cys products enhance the capacity of mitochondrial respiration and alleviate the decline in mitochondrial ATP production during oxygen shortage.

We also tested whether the glycolysis pathway is affected by ΔCM(Cys)∗. It has been widely accepted that glycolytic rates are enhanced in response to hypoxia, with a resulting increase in lactate production. We incubated cardiomyocytes with ΔCM(Cys)∗ and measured the concentration of lactate released into the medium. Incubation in ΔCM(Cys)∗ significantly reduced the production of lactate compared to ΔCM(Cys), both in normoxia and hypoxia (Fig. 4G). Taken together, it is suggested that plasma-irradiated Cys products inhibit metabolic shift towards glycolysis, where ATP demand is met from mitochondria even in hypoxic conditions.

To examine whether sulfide catabolism by SQOR is involved in ΔCM(Cys)∗-mediated recovery of energy metabolism, we performed RNAi knockdown towards SQOR under hypoxia and measured mitochondrial membrane potential (Fig. 4H). Knockdown of SQOR significantly blunted the increase in mitochondrial membrane potential by ΔCM(Cys)∗ (Fig. 4H). Furthermore, overexpression of SQOR under hypoxia significantly increased mitochondrial membrane potential, shown by TMRE fluorescent signal in cardiomyocytes, partially mimicking the effect of ΔCM(Cys)∗ (Fig. 4I). These results suggest that the effect of ΔCM(Cys)∗ in mitochondrial function under hypoxia is mediated by SQOR.

### 2.6 Plasma-irradiated Cys products improve cardiac dysfunction after I/R injury

**Fig. 5:**
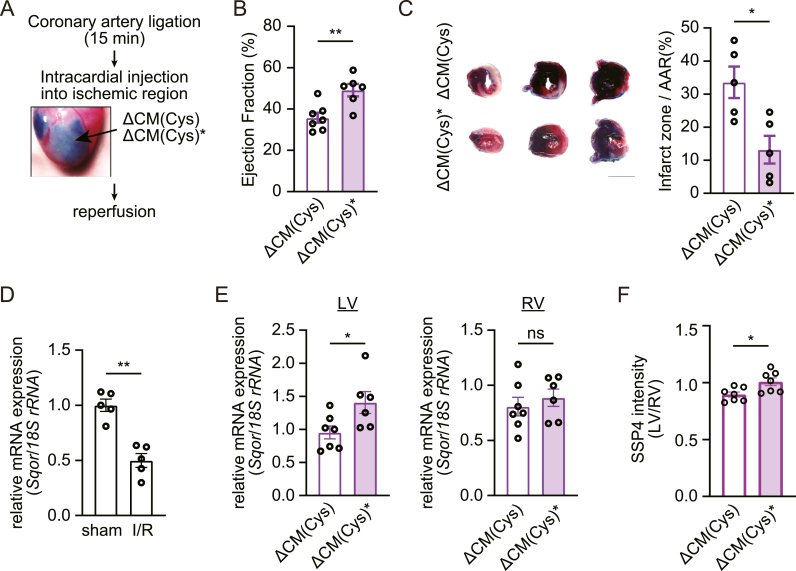
Cardioprotective effect of plasma-irradiated Cys products in ischemia/reperfusion mice model. (**A**) Diagram of intracardial injection of plasma-irradiated Cys medium in ischemia/reperfusion (I/R) model. Dorsal side of the mouse heart was injected with Evans Blue solution. ΔCM(Cys)∗, plasma-irradiated methionine and cysteine-free DMEM supplemented with cysteine. (**B**) Measurement of ejection fraction following I/R (n = 7 mice for ΔCM(Cys), n = 6 mice for ΔCM(Cys)∗). (**C**) Infarcted zone/area at risk (AAR) ratio following I/R (n = 5 mice per treatment). (**D**) Relative mRNA expression level of Sqor genes in left ventricular from sham or I/R mouse heart (n = 5 mice per treatment). (**E**) Relative mRNA expression level of Sqor genes in left ventricular (LV) or right ventricular (RV) from I/R mouse heart (n = 7 mice for ΔCM(Cys), n = 6 mice for ΔCM(Cys)∗). (**F**) Quantification of SSP4 fluorescence intensity in heart lysate. SSP4 intensity in LV was normalized by that in RV (n = 7 mice per treatment). Data are shown as the means ± SEM. ∗P < 0.05, ∗∗P < 0.01 by unpaired *t*-test. (For interpretation of the references to color in this figure legend, the reader is referred to the Web version of this article.)

We next examined whether ΔCM(Cys)∗ has a cardioprotective role in vivo. We intracardially injected ΔCM(Cys)∗ into the left ventricular muscle during ischemia before reperfusion (Fig. 5A). Left ventricular contractile function of the ΔCM(Cys)∗-injected group after 24 h of reperfusion was significantly improved compared to the ΔCM(Cys)-injected group (Fig. 5B–Supplementary Table 1). Furthermore, evaluation of infarct size following reperfusion revealed significant suppression of myocardial death in ΔCM(Cys)∗-treated hearts compared to ΔCM(Cys), as assessed by TTC-Evans Blue staining (Fig. 5C). Myocardial I/R stress itself downregulated mRNA expression of SQOR (Fig. 5D), implying disruption of sulfide catabolism. Importantly, ΔCM(Cys)∗ treatment improved mRNA expression of SQOR in the left ventricular (injected) side (Fig. 5E), suggesting improved sulfide catabolism. Consistent with this, supersulfide level in ΔCM(Cys)∗-treated heart lysate measured by SSP4 was increased compared with ΔCM(Cys)-treated hearts (Fig. 5F). These results suggest that ΔCM(Cys)∗ exerts a cardioprotective effect in vivo heart failure model improving sulfide catabolism via SQOR.

### 2.7 Comparison of plasma-irradiated Cys products with inorganic supersulfide donor

**Fig. 6:**
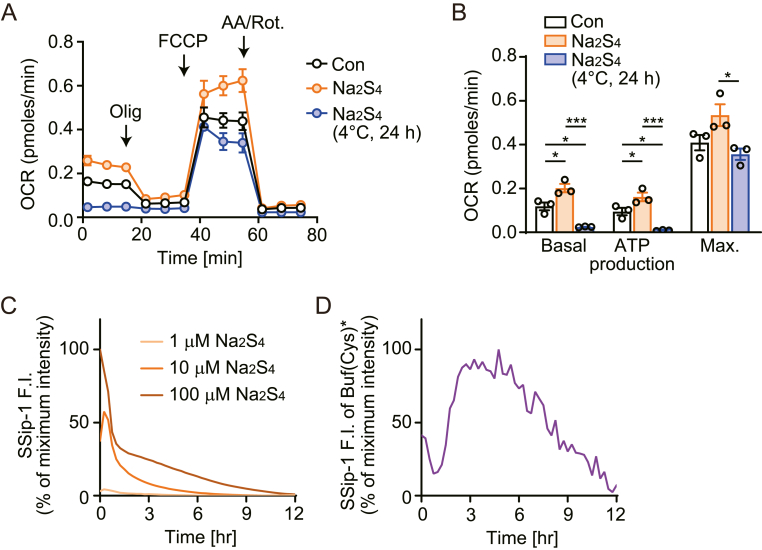
Comparison of supersulfide stability between plasma-irradiated Cys products and inorganic Na_2_S_4_. (**A**) Real-time OCR measurements of cardiomyocytes cultured in the presence (or absence) of freshly prepared or stored (4 °C, 24 h) Na_2_S_4_ by Seahorse extracellular flux analyzer under normoxia. Cells were treated with oligomycin (Olig.), FCCP, rotenone (Rot.) and antimycin A (AA). (**B**) Measurements of OCR for different respiration modes (n = 3 independent experiments). (**C**) Changes in SSip-1 fluorescence intensity in indicated concentrations of Na_2_S_4_ solution. The fluorescence intensity in Na_2_S_4_ was normalized by that in vehicle (n = 3 independent experiments). (**D**) Changes in SSip-1 fluorescence intensity in plasma-irradiated cysteine-containing carbonate buffer (Buf (Cys)∗). The fluorescence intensity in Buf (Cys)∗ was normalized by that in Buf (Cys) (n = 3 independent experiments). Data are shown as the means ± SEM. ∗P < 0.05, ∗∗P < 0.01, ∗∗∗P < 0.001 by one-way ANOVA.

We checked whether inorganic supersulfide donor can mimic the effects of ΔCM(Cys)∗. We previously reported that treatment of cardiomyocytes with freshly prepared Na_2_S_3_ attenuated cardiac dysfunction induced by electrophilic exposure [18]. Freshly prepared Na_2_S_4_ facilitated mitochondrial energy metabolism (Fig. 6A and B). ΔCM(Cys)∗ that is incubated at 4 °C for 24 h increased mitochondrial energy metabolism (Fig. 4D). However, when Na_2_S_4_ was incubated at 4 °C for 24 h, inhibition of oxygen consumption was observed (Fig. 6A and B). This was likely to be induced by sulfide accumulation because Na_2_S_4_ rapidly releases H_2_S (Supplementary Fig. 4). Consistent with this, supersulfide level in Na_2_S_4_ solution measured using SSip1 acutely diminished (Fig. 6C). On the other hand, supersulfide level in Buf (Cys)∗ was stable during 3–6 h after preparation (Fig. 6D) and little amount of H_2_S was detected in Buf (Cys)∗ (Supplementary Fig. 7). These results suggest that plasma-irradiated Cys products are more stable, have less H_2_S generation, and therefore a safer compound than inorganic supersulfide donors. That may propose plasma-irradiated Cys as a new therapeutic agent for cardiovascular diseases.

### 2.8 Cys plasma irradiation in cardiomyocytes is associated with profound alterations in gene expression

**Fig. 7:**
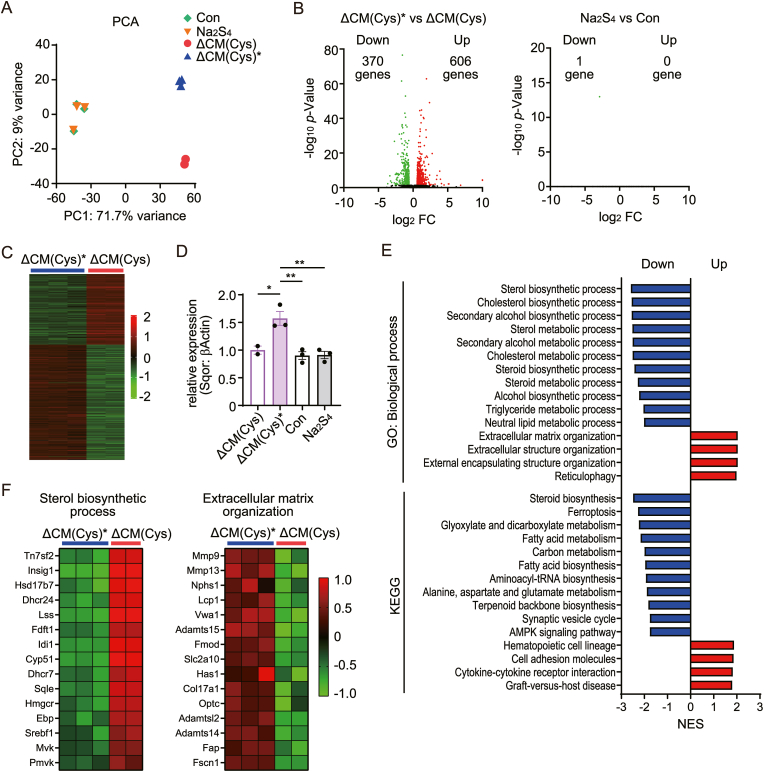
RNA-seq analysis of cardiomyocytes treated with vehicle, Na_2_S_4_, ΔCM(Cys) or ΔCM(Cys)∗ (**A**) Principal component analysis (PCA) of the transcriptome of four different samples. The different color symbols indicated the four different samples. ΔCM(Cys)∗, plasma-irradiated methionine and cysteine-free DMEM supplemented with cysteine. (**B**) Volcano plot of genes differentially expressed in ΔCM(Cys)∗ and ΔCM(Cys) medium (Left) or Na_2_S_4_ and Con (Right). The genes differentially expressed in ΔCM(Cys)∗ or Na_2_S_4_ were plotted in green for downregulated genes and red for upregulated genes. (**C**) Heatmap of gene expression between ΔCM(Cys)∗ and ΔCM(Cys). Genes with significant differences of p < 0.05 were shown. (**D**) The relative expression level of Sqor genes was shown. Data are shown as the means ± SEM. ∗P < 0.05, ∗∗P < 0.01 by one-way ANOVA. (**E**) The plot of GSEA signatures from RNA-seq data between ΔCM(Cys)∗ and ΔCM(Cys) ranked by normalized enrichment score (NES). Negative NES was depleted signature in ΔCM(Cys)∗. Positive NES was enriched signature in ΔCM(Cys)∗. Two different gene sets (gene ontology: biological process and KEGG) were used. (**F**) Heatmap of top 15 enrichment genes for Sterol biosynthetic process and Extracellular matrix organization. (For interpretation of the references to color in this figure legend, the reader is referred to the Web version of this article.)

To compare biological effects of ΔCM(Cys)∗ and Na_2_S_4_, the transcriptional changes in cardiomyocytes treated with vehicle (Con), Na_2_S_4_, ΔCM(Cys) and ΔCM(Cys)∗ were analyzed by RNA-seq analysis. Principal component analysis (PCA) demonstrated the separation of ΔCM(Cys) and ΔCM(Cys)∗, but not Con and Na_2_S_4_ (Fig. 7A). Differentially expressed gene (DEG) analysis showed that 606 genes were upregulated and 370 genes were downregulated with false discovery rate (FDR) ≤0.05 and fold change (FC) ≥1.5 in ΔCM(Cys)∗ compared to ΔCM(Cys), whereas only one gene was downregulated in Na_2_S_4_ compared to Con (Fig. 7B and C). Consistent with qPCR experiment (Fig. 3D), the expression of SQOR mRNA was increased in ΔCM(Cys)∗ but not Na_2_S_4_ (Fig. 7D). Gene set enrichment analysis (GSEA) between ΔCM(Cys)∗ and ΔCM(Cys) using two different gene sets (gene ontology: biological process and KEGG) revealed that sterol biosynthetic and metabolic pathways are downregulated and extracellular matrix organization pathways are upregulated in ΔCM(Cys)∗ (Fig. 7E and F). Additionally, ΔCM(Cys)∗ treatment altered gene expressions related to ferroptosis and cytokines (Supplementary Figs. 8 and 9). These results suggest that ΔCM(Cys)∗ has distinctly different biological effects compared to ΔCM(Cys) and Na_2_S_4_.

## 3 Discussion

Our results inform non-thermal plasma irradiation as a novel method to activate supersulfide synthesis through SQOR in cardiomyocytes. Although H_2_S donors have been proven beneficial for ischemia injury in animal models [42,43], there are still challenges for clinical applications: H_2_S is potentially toxic and has a small therapeutic window, with a complex reaction network in which the bioactivity of H_2_S is difficult to control [44]. Moreover, thanks to recent advances in supersulfide-specific quantification methods developed by several groups [10,11,36], it has become increasingly evident that supersulfides are the bona fide mediators of redox signaling involved in lifespan extension across species [11,12] and have beneficial effects on the cardiovascular system [17,18,33,34,45]. Considering the unique feature of plasma-mediated chemical reactions, we posited that plasma irradiation might enable to promote endogenous supersulfide synthesis from non-H_2_S biomolecules, which potentially circumvents the cytotoxicity of H_2_S. In this study, we first found contrasting bioactivity in plasma-irradiated Cys vs. Met. ΔCM(Cys)∗ attenuated cardiomyocyte death induced by OGD/R stress, whereas ΔCM(Met)∗ exacerbated it (Fig. 1F and G). Also, an increase in hydropersulfides was found in ΔCM(Cys)∗, but not in ΔCM(Met)∗ (Fig. 2A and B). Therefore, our results suggest that ΔCM(Met)∗ contains toxic molecules. For example, methionine metabolite in bacteria during fermentation includes dimethyl trisulfide (Me-SSS-Me) and dimethyl tetrasulfide (Me-SSSS-Me) [46]. It has been demonstrated that these metabolites induce apoptosis in certain cell lines and that the toxicity is completely suppressed by the addition of GSH [47]. Therefore, it is possible that the dissociation/reconstruction of molecular bonds in Met by plasma irradiation gives rise to these toxic metabolites. Further studies will be needed to identify toxic components in ΔCM(Met)∗.

Identifying a bona fide biological component that causes plasma-irradiated cardioprotection is one of the most interesting aspects. Intensive research on plasma biology has elucidated various aspects of chemical reaction networks in plasma-irradiated solutions [48]. We found that not only plasma-irradiated DMEM but also Cys in carbonate buffer (containing NaCl, NaHCO_3_ and glucose) has cardioprotective activity (Fig. 1 and Supplementary Figs. 4 and 6). Plasma treatment of solutions containing inorganic salts generates ROS (e.g. OH radicals) and reactive nitrogen species (RNS) in liquid phase and finally results in the accumulation of long-lived species such as H_2_O_2_, NO_2_^−^ and NO_3_^−^ [48,49]. Glucose is converted into its oxidation products such as gluconic acid and glucuronic acid [50]. More importantly, product analysis of plasma-irradiated Cys solutions has been previously reported [51,52]. Plasma treatment of Cys in pure water generates cysteine sulfonic acid, cystine and cysteine fragments as major products and oxidized cysteine derivatives and S-nitrosylated cysteines as minor products [51]. Although previous product analysis has not revealed the production of CysSSH, we found that CysSSH formation is increased in Buf (Cys)∗ using quantitative mass spectrometry analysis highly optimized for supersulfides (Fig. 2B). In the cell, CARS2 is the major enzyme to synthesis CysSSH from two Cys. In detail, CARS2 catalyzes the cleavage sulfur atom from donor Cys and its transfer to acceptor Cys for CysSSH formation [8]. Plasma irradiation may non-enzymatically catalyze CysSSH formation by sulfur transfer reaction. Another possibility is that plasma irradiation catalyzes CysSSH formation from cystine through CS lyase-like reaction [10]. The half-life of supersulfides themselves is short [53]. Therefore, CysSSH and other supersulfides would be constantly synthesized and decomposed by reacting with long-lived species in plasma-irradiated solutions. Administration of plasma-irradiated Cys products rapidly increased the intracellular supersulfide levels in cardiomyocytes (Fig. 2C), suggesting the direct transfer of supersulfides into the cells through transporters or channels. Mass spectrometry analysis also identified further oxidation of supersulfides to sulfite and thiosulfate in Buf (Cys)∗ (Supplementary Fig. 3). Because the extracellular application of thiosulfate induces supersulfide formation across species and extends lifespan in an H_2_S-independent manner [11,54], the process of cysteine oxidation triggered by plasma irradiation may also contribute to supersulfide formation in the cell. We have previously reported that the maintenance of intracellular supersulfide levels at high level in cardiomyocytes plays a pivotal role in resistance to various stresses such as hypoxia, mechanical load and electrophiles [[16], [17], [18],^34^]. While further studies will be needed to understand the whole reaction network in plasma-irradiated Cys solutions, our data suggest that the mixture of cysteine metabolites produced by plasma irradiation works as a whole against ischemia/reperfusion stress.

It has been reported that plasma irradiation of artificially high concentrations (100 mM) of Cys triggers the inhibition of proliferation and migration of human keratinocytes, whereas plasma-irradiated Cys products at physiological (2 mM) Cys concentrations have no impact on phenotypes [52]. We found that treatment of cardiomyocytes with plasma-irradiated Cys at physiological Cys concentration showed no cytotoxic effects and improved mitochondrial bioenergetics under hypoxia (Fig. 4). In most mammalian cells, oxygen is an essential electron acceptor in mitochondrial ETC. Indeed, prolonged oxygen shortage results in stalling of electron-coupled reactions and impaired ETC. On the other hand, recent studies suggest that persulfides may serve as an alternative electron acceptor [8]. In this additional electron-coupled reaction, persulfides are reduced to sulfides by accepting electrons from ETC, and sulfides are oxidized back to persulfides by SQOR [14]. This redox cycle not only supports oxygen-dependent bioenergetics, but plays an important role in developing resistance to hypoxia [14]. In the current investigation, we found that ΔCM(Cys)∗ treatment altered the expression levels of SQOR, which can prioritize sulfide consumption to synthesize supersulfides, leading to maintained mitochondrial membrane potential during oxygen shortage (Fig. 3, Fig. 4). Additionally, RNA-seq analysis found that ΔCM(Cys)∗ treatment altered the expression levels of various genes including sterol metabolism, ferroptosis and cytokine (Fig. 7). Ferroptosis-related genes were mainly down-regulated by ΔCM(Cys)∗ treatment, suggesting that plasma-irradiated Cys products mediate attenuation of ferroptosis. A wide variety of studies identified that ferroptosis has emerging roles in myocardial I/R injury [55,56]. Moreover, cholesterol is involved in ferroptosis resistance in several cells [57,58]. Since supersulfides are reported to negatively regulate ferroptosis, it is plausible that ΔCM(Cys)∗ downregulates ferroptosis-related gene pathways [59]. Ferroptosis may be an alternative target for plasma-irradiated Cys products. While further studies will be required to understand the cardioprotective mechanism of ΔCM(Cys)∗, it is possible that various metabolic intermediates synthesized by plasma irradiation activate cardioprotective events by affecting the expression and activity of related genes and proteins.

Finally, the current study demonstrates that plasma irradiation of thiol-containing amino acid Cys results in the generation of supersulfides such as CysSSH, and that its application to cardiomyocytes prevented mitochondrial dysfunction by maintaining mitochondrial bioenergetics. Moreover, plasma-irradiated Cys products promoted sulfide catabolism and this also would contribute to maintaining mitochondrial function. Biased synthesis towards supersulfides rather than H_2_S and other sulfides by plasma irradiation may minimize sulfide toxicity and provide a novel therapeutic approach to ischemia-reperfusion injury.

## 4 Methods

### 4.1 Animals

All experiments using animals were approved by an Ethics Committee of National Institutes of Natural Sciences and carried out in accordance with their guidelines. Sprague-Dawley (SD) rats were purchased from Japan SLC Inc. C57BL6J mice were from The Jackson Laboratory Japan Inc. We used 8- to 16- week-old male mice in all studies.

### 4.2 Reagents

All methods were performed in accordance with the guidelines and regulations of chemical substance management and approved by the committees of chemical substance management at the National Institutes of Natural Sciences. Dulbecco's modified Eagle medium (DMEM) without l-methionine and l-cystine (Sigma-Aldrich) was used to dissolve 0.2 mM of l-cysteine (Wako) or l-methionine (Wako). SSip-1 DA was purchased from Goryo Chemical, Inc.

### 4.3 Cell culture and hypoxia

Neonatal rat cardiomyocytes (NRCMs) were isolated from 2-day-old SD rats, and cultured in DMEM (low glucose) containing 10 % (vol/vol) fetal bovine serum (FBS) as described previously [60]. To expose the cells to hypoxic condition, cells were transferred into a multigas incubator (Panasonic) containing 1 % O_2_.

### 4.4 Indirect plasma irradiation to cells

Culture medium or buffer solution was irradiated with the non-thermal plasma jet using helium as the carrier gas, and then applied to cultured cells, as described previously [26,49,61]. Briefly, the flow rate of helium gas was set to 3 L/min by the mass flow controller (Fujikin). The applied voltage and frequency were 7–9 kV and 8 kHz, respectively. Cysteine or methionine (2 mM) in DMEM (-Cys, -Met) or carbonate buffer (110 mM NaCl, 44 mM NaHCO_3_ and 250 mM glucose) was irradiated with the non-thermal helium plasma jet, and then incubated for 6 h at 37 °C. As a control, Cys solution was incubated. Plasma irradiated solution was diluted to 10-fold with DMEM (-Cys, -Met), and then mixed with 100 μg/mL catalase (Sigma) and 10 mM HEPES for cell treatment. Measurement of H_2_O_2_ concentration in plasma-irradiated solution was conducted by the colorimetric staining probe (WAK-H2O2, Kyoritsu Chemical-Check Laboratory) in the presence or absence of 100 μg/mL catalase.

### 4.5 Cell death assay

Cardiomyocytes were cultured in DMEM (no glucose). For OGD condition, plasma irradiated solution was diluted to 10-fold with DMEM (no glucose) instead of DMEM(-Cys, -Met). Cardiomyocytes cultured in medium with or without plasma irradiation are incubated under hypoxia (1 % O_2_) for 24 h, followed by normoxia (21 % O_2_) for 12 h. Cells were stained with 2 μg/mL propidium iodide (Dojindo) and 2 μg/mL Hoechst 33342 (Dojindo). Stained nuclei were visualized by BZ-X710 fluorescent microscope (Keyence) and counted using ImageJ software. Cell death rate (%) was expressed as a percentage of propidium iodide-positive nuclei/Hoechst-positive nuclei.

### 4.6 Measurement of supersulfides in the liquid phase

FRET-based fluorescent probe QS10 [35] was used for measuring supersulfides in plasma-irradiated solutions. Cys or Met (2 mM) in carbonate buffer was irradiated with the non-thermal helium plasma jet and incubated for 6 h at 37 °C. We applied 1 μM of QS10 to plasma irradiated solutions and recorded fluorescence ratio (CH1: Ex 550 nm and Em 595 nm, CH2: Ex 550 nm and Em 645 nm) by SpectraMax i3 plate reader (Molecular Devices). For quantification, the indicated concentrations of Na_2_S_4_ in carbonate buffer were immediately mixed with QS10, and the fluorescence ratio was recorded. For measurement of hydrogen sulfide (H_2_S), 1 μM of HSip1 was applied and then incubated for 1 h, followed by measurement of green fluorescence (Ex 491 nm, Em 516 nm). Quantification of supersulfides by LC-MS/MS using the HPE-IAM trapping method was conducted according to a previous study [36].

### 4.7 Live cell imaging

For the measurement of intracellular H_2_S and supersulfides, cardiomyocytes were preloaded with 2 μM of SF7-AM and 1 μM of QS10 (including 0.1 % DMSO and 0.02 % Pluronic F127), respectively. After loading for 15 min at 37 °C, cells were washed with HBSS and imaged by BZ-X710 fluorescent microscope (Keyence) or SP8 confocal microscope (Leica). For quantification of fluorescence intensity of SF7-AM, Hoechst staining was used to ensure that cells were focalized. For ratiometric analysis based on QS10 live cell imaging (CH1 and CH2), LAS X (Leica) was used. Fluorescence probe SSip-1 was also used to measure supersulfides. Cells were preloaded with 1 μM of SSip-1 (including 0.1 % DMSO, 1 mg/ml BSA and 0.02 % Pluronic F127) for 30 min. Green fluorescence (Ex 488 nm, Em 525 nm) was imaged by BZ-X710.

For FRET-based measurement of mitochondrial ATP concentration, mit-ATeam 1.03 plasmid was transfected into cardiomyocytes [27] by Lipofectamine™ 3000 transfection reagent (Thermo Fisher Scientific). Cells were observed under a confocal microscope (Leica TCS SP8), using HC PL APO CS2 63x/1.20 water immersion objective lens. 458 laser was activated and fluorescence was detected by PMT1 (468 nm–505 nm) and PMT3 (524 nm–601 nm), respectively. Pixel-by-pixel calculation of YFP/CFP emission ratio in each cell was done by LAS X software (Leica).

### 4.8 Mitochondrial energy metabolism

For the analysis of mitochondrial membrane potential, cardiomyocytes were incubated with JC-1 dye (Invitrogen) for 30 min at 37 °C. After several washes, fluorescent images were acquired by BZ-X710 fluorescent microscope (Keyence). The mitochondrial membrane potential was expressed as a fold change of red (Ex 579 nm, Em 599 nm)/green (Ex 485 nm, Em 516 nm) fluorescence intensity recorded by SpectraMax i3 plate reader (Molecular Devices).

Oxygen consumption rate (OCR) was measured using Seahorse XF HS Mini Extracellular Flux Analyzer (Agilent). For Flux Analyzer experiment, ΔCM(Cys)∗ medium was incubated at 4 °C for 24 h instead of 37 °C for 6 h. NRCMs that were plated on microplates at a density of 15,000 cells per well were cultured in ΔCM(Cys)∗ medium for 24 h. XF HS mini assay media supplemented with 25 mM glucose, 1 mM Pyruvate, and 2 mM of l-glutamine in DMEM was prepared and used to prepare cellular stress reagents to provide the following final concentrations: 1.5 μM Oligomycin, 2 μM FCCP, 2.5 μM Antimycin A and 2.5 μM Rotenone. OCR for mitochondrial respiration/OXPHOS was measured for 3 min with 3 min of mixing and 2 min of the waiting period. A total of 12 measurements were taken. OCR was normalized by cell number and expressed as pmoles oxygen/min/10^4^ cells. OCR of cardiomyocytes was also measured by MitoXpress Xtra oxygen consumption assay kit (Agilent), using a time-resolved fluorescence module installed in SpectraMax i3 (Molecular Devices), according to the manufacturer's instructions. NRCMs that were plated on 96-well plates were cultured in ΔCM(Cys)∗ medium under normoxia or hypoxia for 24 h. The medium was changed to DMEM with the oxygen sensor probe as soon as cells were exposed to oxygen. Data were represented as fold change of slope determined by linear regression using GraphPad Prism 8.0 software (GraphPad).

### 4.9 Measurement of cellular metabolites

Lactate concentration in the culture medium was measured by Lactate Assay Kit-WST (Dojindo) according to the manufacturer's instructions. Intracellular NAD^+^/NADH ratio was measured by NAD/NADH-Glo™ Assay (Promega) according to the manufacturer's instructions, where luminescence intensities derived from NAD^+^ and NADH were separately recorded from the same sample. Data were expressed as fold change of NAD^+^/NADH ratio.

### 4.10 Murine model of myocardial ischemia-reperfusion injury

Left coronary artery ligation in mice was performed as described previously [39], with several modifications: we performed 15 min of ischemia followed by 24 h of reperfusion. Reperfusion was confirmed by the reversal of color changes of the vessel and cardiac tissue in the left ventricle. When procedural abnormalities were observed (e.g. incomplete ischemia or incomplete reperfusion), animals were excluded from the analysis. ΔCM(Cys)∗ or ΔCM(Cys) medium was immediately administered intracardially after ischemia. Cardiac function was then monitored by echocardiography using Vevo 3100 imaging system (VisualSonics). Assessment of infarct size was conducted as previously described [62].

### 4.11 Detection of supersulfides in cardiac tissue

After the intracardial injection of ΔCM(Cys)∗ or ΔCM(Cys) medium, the heart was harvested and divided into left ventricle (LV) and right ventricle (RV). The LV and RV were homogenized in lysis buffer (20 mM HEPES, 100 mM NaCl, 3 mM MgCl_2_, 1 % SDS). The tissue lysate was centrifuged at 16,000 g for 3 min and the supernatant was collected. The supernatant was mixed with SSP4 (Dojindo, final concentration: 10 μM), and fluorescence intensity (Ex 482 nm, Em 515 nm) was measured using SpectraMax i3 plate reader.

### 4.12 Quantitative reverse transcript polymerase chain reaction (qRT-PCR)

Total RNA was isolated from isolated cardiomyocytes using ReliaPrep™ miRNA Cell and Tissue Miniprep System (Promega) or from mouse heart tissues using RNeasy® Fibrous Tissue Mini Kit (QIAGEN) following the manufacture's instruction. Complementary DNA (cDNA) was synthesized by reverse transcription using ReverTra Ace® qPCR RT Kit (TOYOBO). qRT-PCR was performed using KAPA SYBR FAST qPCR Kits following the manufacture's instruction. The sequence of the primers we used: rat Sqor forward 5′-AGTTGGAGCGGAGAATGTGG-3′, reverse 5′-CTGCACACCGGATGGAATCA-3’; mouse Sqor forward 5′-CTTCCCAAACACTCCGGTGA-3′, reverse 5′-TGGGTCGCTTTCCAGTCTTC-3’; rodent 18S rRNA forward 5′-ATTAATCAAGAACGAAAGTCGGAGGT-3′, reverse 5′-TTTAAGTTTCAGCTTTGCAACCATACT-3’.

### 4.13 Library construction and RNA sequencing

NRCMs were cultured in medium containing ΔCM(Cys)∗, ΔCM(Cys) or 100 nM Na_2_S_4_ for 24 h, and total RNA was isolated as described above. The isolated RNA's quality check, library synthesis and NGS sequencing were performed by BGI genomics. The libraries were sequenced on the DNBSEQ platform using the PE100 strategy. HISAT was used to align the filtered data to the reference genome (GCF_000001895.5_Rnor_6.0).

### 4.14 Differential gene expression analysis and pathway enrichment analysis

RNA-seq count data were analyzed using the iDEP.96 (Integrated Differential Expression and Pathway analysis) [63]. Basically, the default setting was used for analysis. DEGs from two group comparisons (ΔCM(Cys)∗ vs ΔCM(Cys) or Na_2_S_4_ vs Con) were obtained by the DESeq2 with fold-change >1.5 or < 0.67 and threshold of false discovery rate (FDR) < 0.05. For pathway enrichment analysis, gene set enrichment analysis (GSEA) was performed. The analysis used gene ontology biological process (GO BP) and Kyoto encyclopedia of genes and genomes (KEGG) gene set databases.

### 4.15 Statistical analysis

All results were presented as the mean ± S.E.M. from at least three independent experiments. Statistical comparisons were carried out using unpaired *t* tests for two-group comparisons or using one-way ANOVA followed by Tukey's *post hoc* test for multiple comparisons. Values of P < 0.05 were considered to be statistically significant.

## CRediT authorship contribution statement

**Akiyuki Nishimura:** Writing – review & editing, Writing – original draft, Funding acquisition, Data curation, Conceptualization. **Tomohiro Tanaka:** Writing – original draft, Data curation, Conceptualization. **Kakeru Shimoda:** Writing – review & editing, Data curation. **Tomoaki Ida:** Writing – review & editing, Data curation. **Shota Sasaki:** Writing – review & editing, Resources, Methodology. **Keitaro Umezawa:** Writing – review & editing, Resources. **Hiromi Imamura:** Writing – review & editing, Resources. **Yasuteru Urano:** Writing – review & editing, Resources. **Fumito Ichinose:** Writing – review & editing, Methodology. **Toshiro Kaneko:** Writing – review & editing, Resources. **Takaaki Akaike:** Writing – review & editing, Resources, Funding acquisition, Data curation. **Motohiro Nishida:** Writing – review & editing, Writing – original draft, Funding acquisition, Conceptualization.

## Declaration of competing interest

The authors have declared that no conflict of interest exists.

## Data Availability

Data will be made available on request.
