# Supplementary material for: Non-thermal atmospheric pressure plasma-irradiated cysteine protects cardiac ischemia/reperfusion injury by preserving supersulfides
Source: Redox Biol. 2024 Nov 28;79:103445. doi: 10.1016/j.redox.2024.103445 (PMC11663985; doi:10.1016/j.redox.2024.103445)
Supplement: Multimedia component 1 [file mmc1.docx]

**Supplementary Information**

**Non-thermal atmospheric pressure plasma-irradiated cysteine protects cardiac ischemia/reperfusion injury by preserving supersulfides**

**Authors:** Akiyuki Nishimura, Tomohiro Tanaka, Kakeru Shimoda, Tomoaki Ida,

Shota Sasaki, Keitaro Umezawa, Hiromi Imamura, Yasuteru Urano, Fumito Ichinose,

Toshiro Kaneko, Takaaki Akaike and Motohiro Nishida


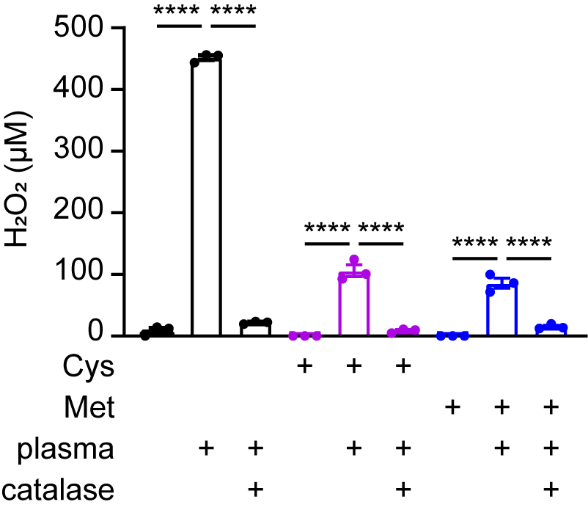


**Supplementary Fig. 1.** **Plasma-dependent hydrogen peroxide (H_2_O_2_) production.**

H_2_O_2_ concentration in plasma-irradiated cysteine and methionine-free DMEM (ΔCM) (black), ΔCM+Cys (Purple) and ΔCM+Met (Blue) was measured using a colorimetric staining probe (n=3 independent experiments). Data are presented as the mean ± SEM. ****P<0.0001 by one-way ANOVA.


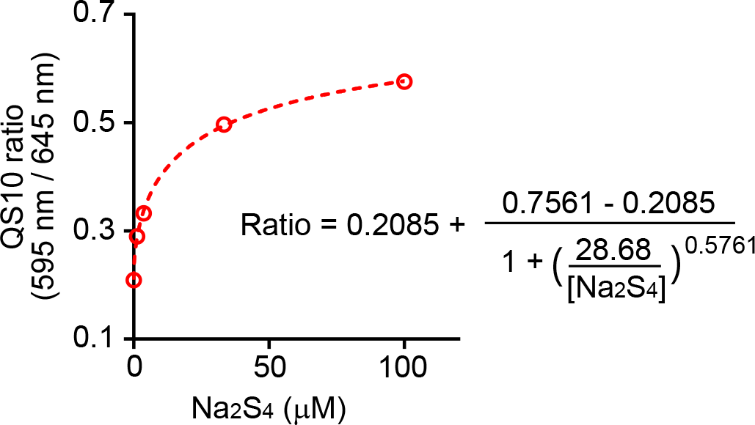


**Supplementary Fig. 2.** **Quantification of supersulfide titer of plasma-irradiated Cys solution**

Dose-response curve of Na_2_S_4_ was measured by QS10 probe fluorescence ratio. Curve fitting was performed with GraphPad Prism. Fluorescence ratio of plasma-irradiated cysteine solution (Fig. 2A) was equivalent to 80 nM of Na_2_S_4_.


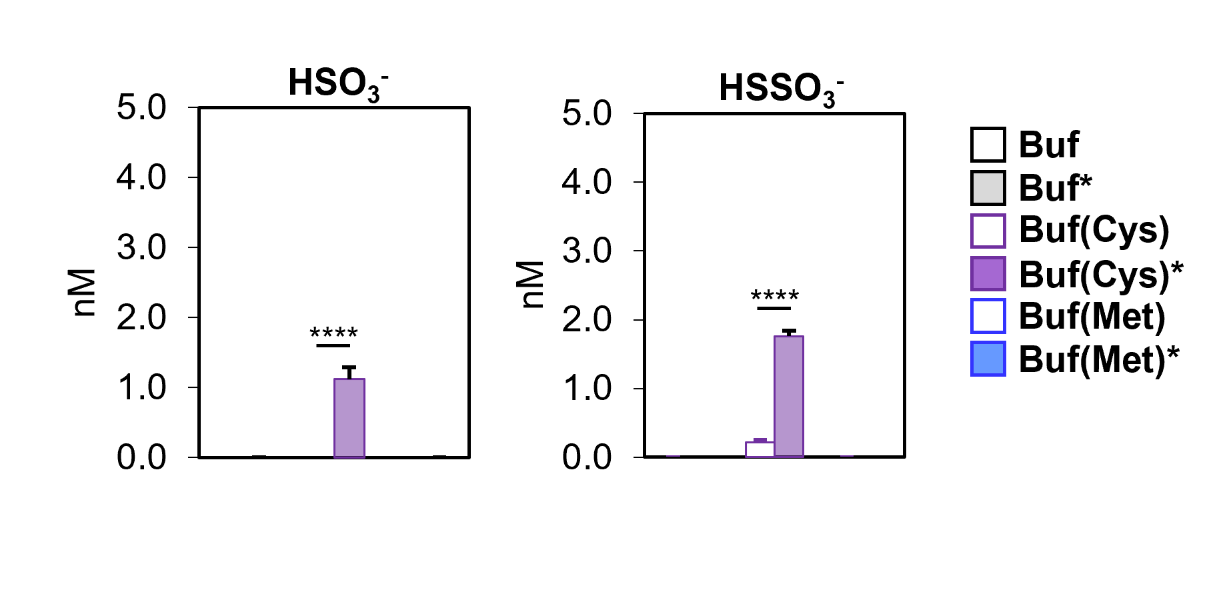


**Supplementary Fig. 3.** **Mass spectrometry analysis of plasma-irradiated Cys solutions.**

Quantitative analysis of HPE-IAM-trapped polysulfides by LC-MS/MS method in plasma-irradiated carbonate buffer supplemented with cysteine or methionine (Buf(Cys)* or Buf(Met)*, respectively) (n=3 independent experiments). Data are presented as the mean ± SEM. ****P<0.0001 by one-way ANOVA.


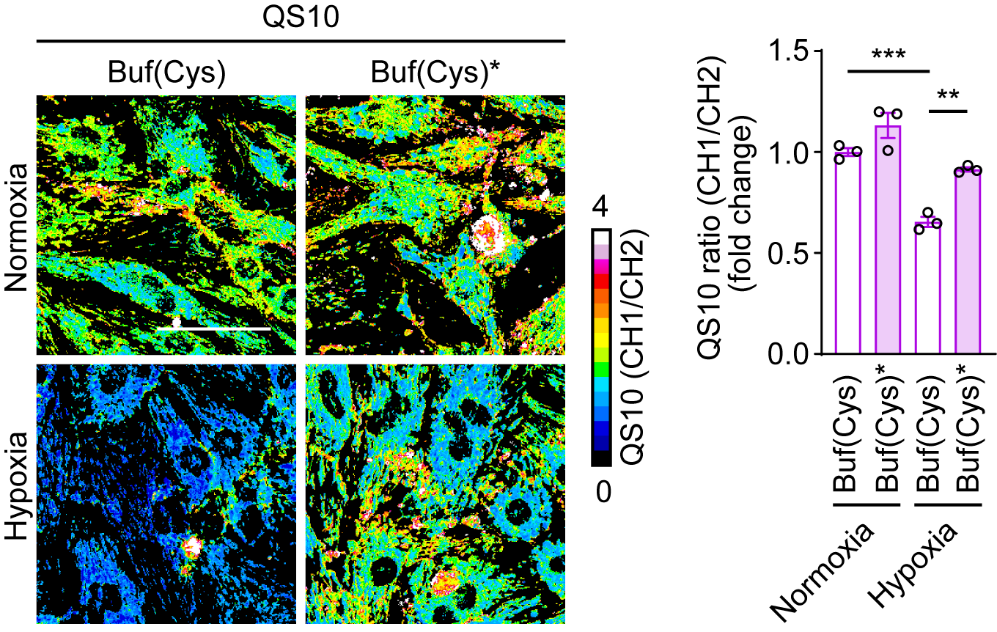


**Supplementary Fig. 4. Effect of plasma-irradiated Cys buffer on hypoxia-mediated supersulfide reduction.**

QS10 (ratiometric pseudocolored) imaging for supersulfides in cardiomyocytes. Cardiomyocytes treated with plasma-irradiated cysteine-containing carbonate buffer (Buf(Cys)*) were cultured under normoxia or hypoxia. Scale bar, 50 μm. Bar graph: Quantification of QS10 CH1/CH2 ratio (n=3 independent experiments). Data are shown as the means ± SEM. **P<0.01, ***P<0.001 by one-way ANOVA.

**
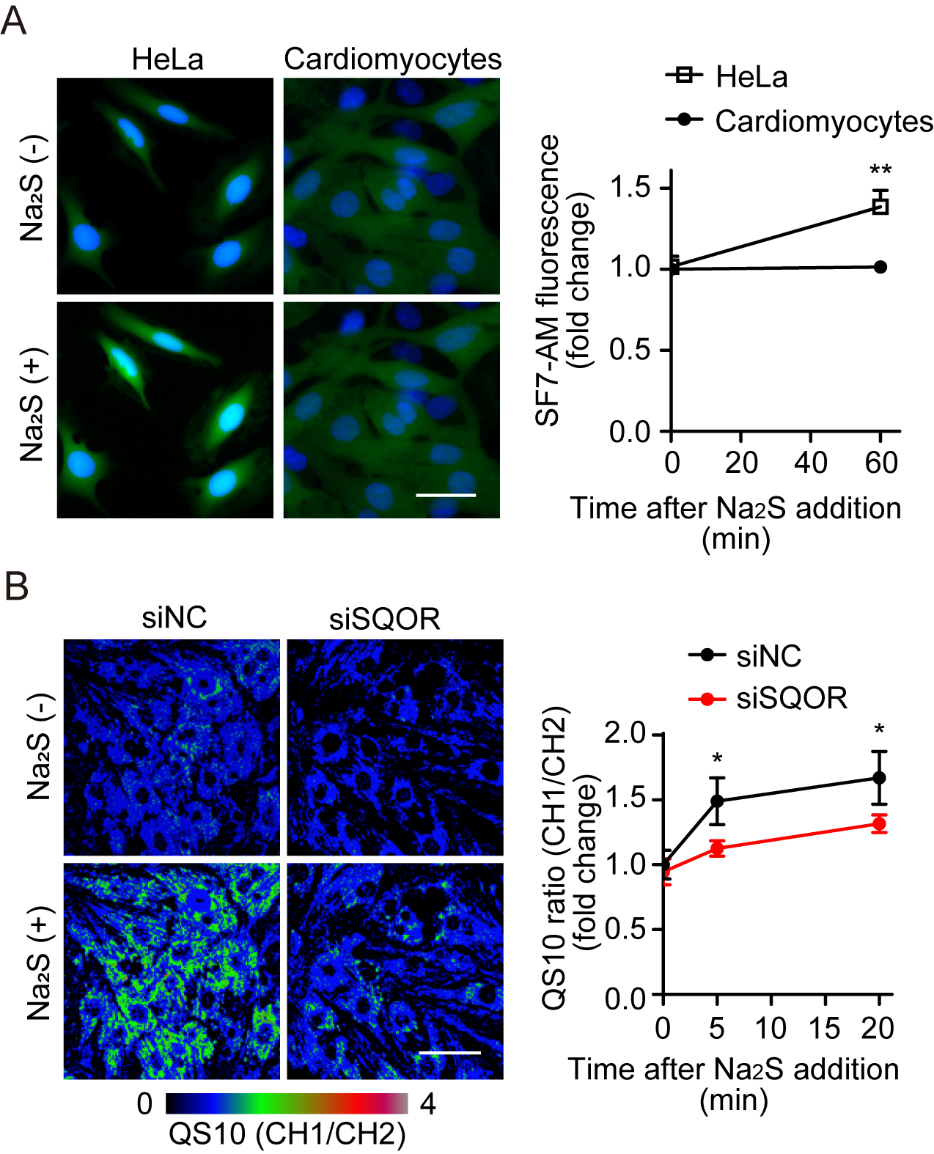
**

**Supplementary Fig. 5. Acute conversion of Na_2_S to supersulfides in cardiomyocytes.**

(**A**) Comparison of H_2_S accumulation after Na_2_S addition between HeLa cells and cardiomyocytes. Cells were preloaded with SF7-AM (green) and Hoechst (blue), following Na_2_S treatment. Scale bar, 40 μm. Right: Time course of SF7-AM fluorescence upon Na_2_S application (n=3 independent experiments). (**B**) The effect of SQOR knockdown on sulfide catabolism. Cardiomyocytes transfected with siRNA for negative control (siNC) or SQOR (siSqor) were preloaded with QS10, following Na_2_S application. Scale bar, 50 μm. Right: Time course of QS10 Ch1/Ch2 ratio upon Na_2_S application (n=3 independent experiments). Data are shown as the means ± SEM. *P<0.05, **P<0.01 by unpaired t test (D).


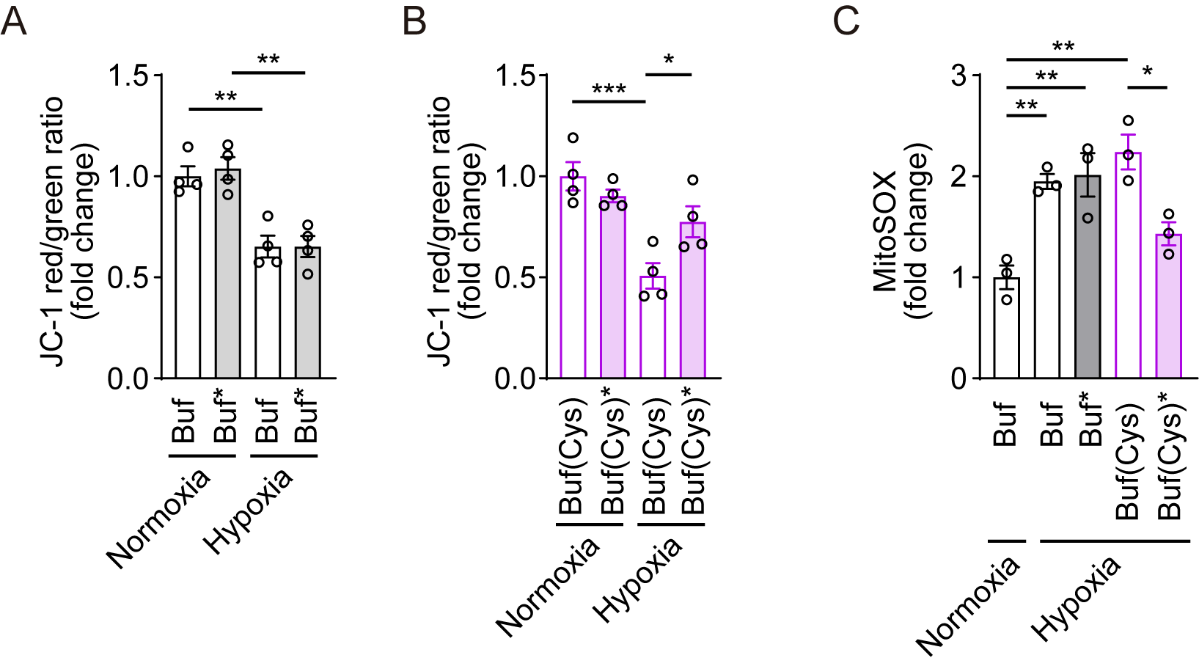


**Supplementary Fig. 6. Effect of plasma-irradiated Cys buffer on mitochondrial functions.**

(**A, B**) Analysis of mitochondrial membrane potential by JC-1 dye. Cardiomyocytes treated with plasma-irradiated carbonate buffer (Buf*) (**A**) or plasma-irradiated carbonate buffer supplemented with cysteine (Buf(Cys)*) (**B**) were cultured under normoxia or hypoxia. JC-1 red/green fluorescence ratio was quantified (n=4 independent experiments). (**C**) Analysis of mitochondrial ROS generation by MitoSOX dye. Cardiomyocytes treated with Buf* or Buf(Cys)* were cultured under Nor or Hyp. MitoSOX intensity was quantified (n=3 independent experiments). Data are shown as the means ± SEM. *P<0.05, **P<0.01, ***P<0.001 by one-way ANOVA.


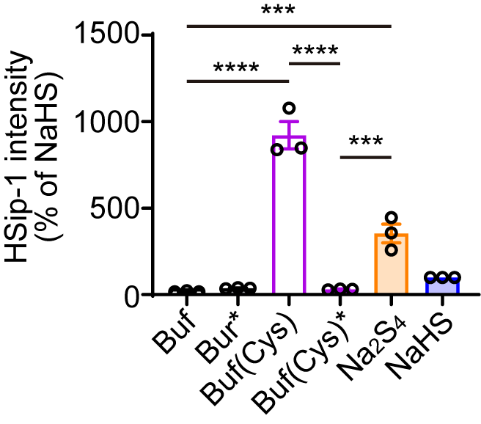


**Supplementary Fig. 7. Generation of H_2_S in plasma irradiated cysteine buffer and Na_2_S_4_.**

H_2_S detection probe HSip-1 fluorescence intensity in plasma-irradiated carbonate buffer supplemented without or with cysteine (Buf* or Buf(Cys)*), Na_2_S_4_ and NaHS was measured (n=3 independent experiments). Data are presented as the mean ± SEM. ***P<0.001, ****P<0.0001 by one-way ANOVA.


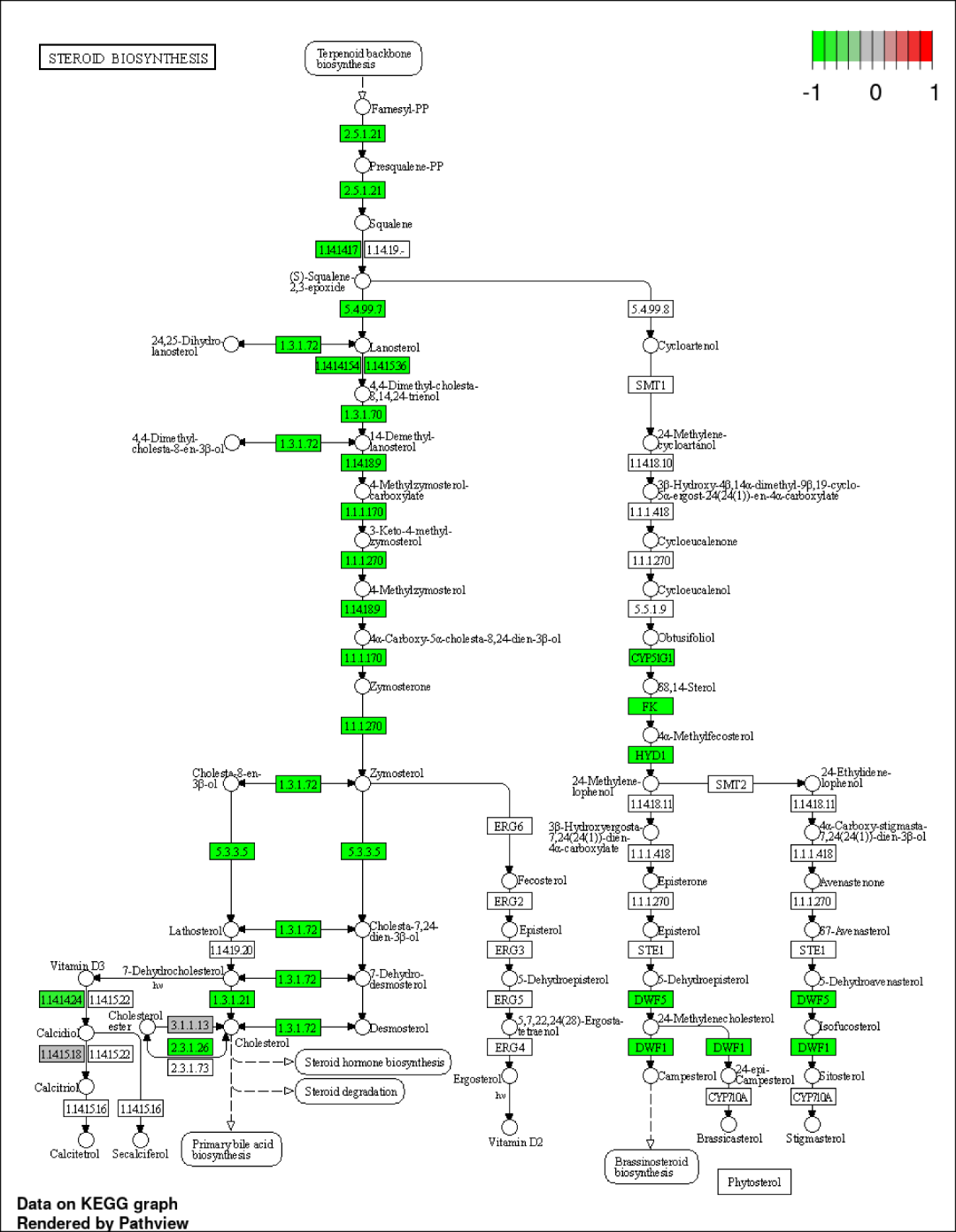


**Supplementary Fig. 8. KEGG enrichment of DEGs between ΔCM(Cys)* and ΔCM(Cys) in steroid biosynthesis.**

Green and red boxes show downregulated and upregulated genes in ΔCM(Cys)* compared to ΔCM(Cys). ΔCM(Cys), methionine and cysteine-free DMEM supplemented with cysteine; ΔCM(Cys)*, plasma-irradiated ΔCM(Cys).


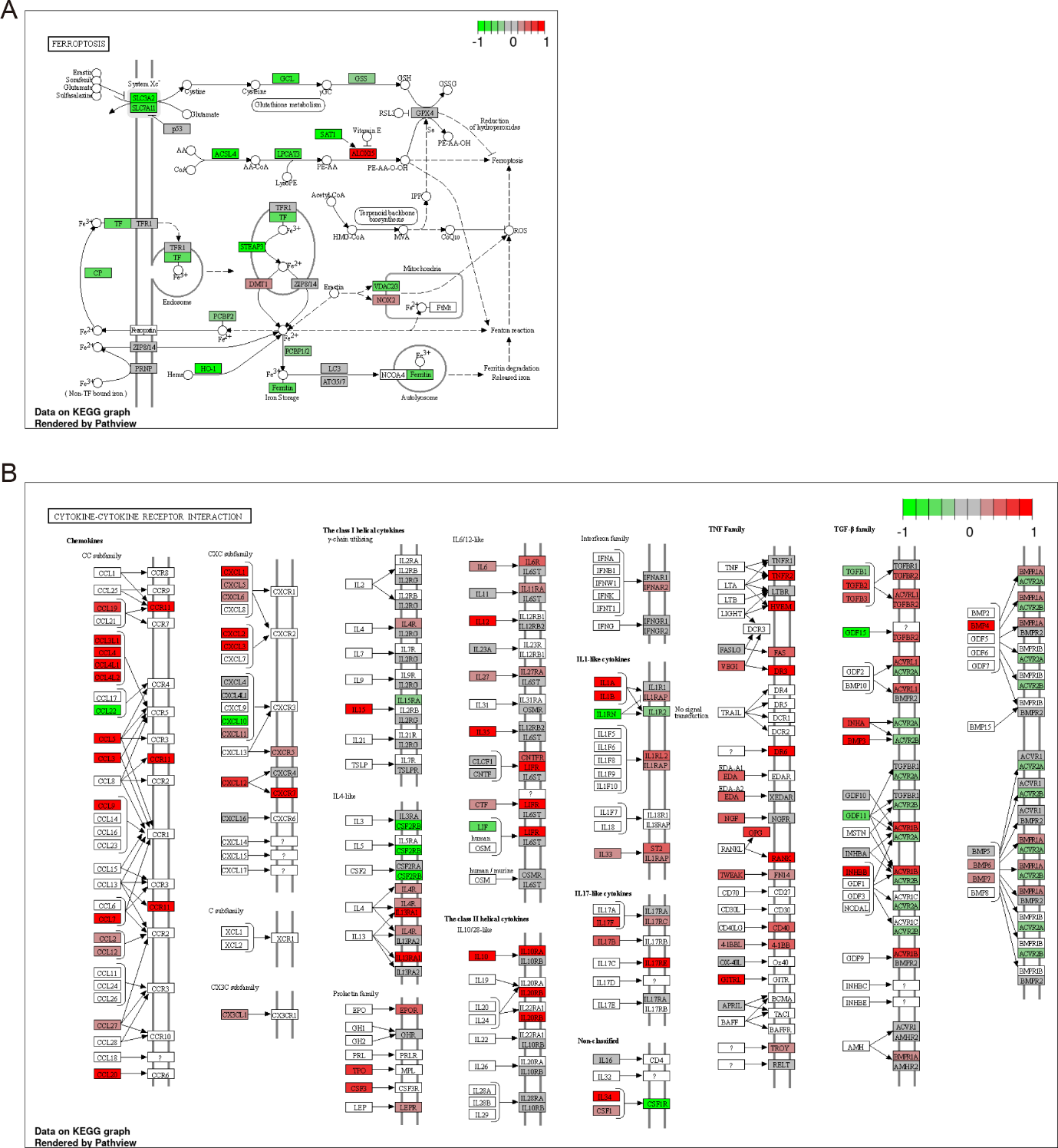


**Supplementary Fig. 9. KEGG enrichment of DEGs between ΔCM(Cys)* and ΔCM(Cys) in ferroptosis and cytokine-cytokine receptor interaction.**

(**A**) Ferroptosis pathway. (**B**) Cytokine-cytokine receptor interaction. Green and red boxes show downregulated and upregulated genes in ΔCM(Cys)* compared to ΔCM(Cys). ΔCM(Cys), methionine and cysteine-free DMEM supplemented with cysteine; ΔCM(Cys)*, plasma-irradiated ΔCM(Cys).

**Supplementary Table 1. Cardiac parameters of plasma-irradiated Cys medium injected-I/R mice measured by echocardiography.**

|  | Sham  (n=5) | ΔCM(Cys) I/R (n=7) | ΔCM(Cys)^*^ I/R  (n=6) |
| --- | --- | --- | --- |
| volume;s (μL) | 20.5 ± 2.5 | 32.8 ± 2.4^**^ | 20.6 ± 2.3^††^ |
| volume;d (μL) | 46.4 ± 3.1 | 50.9 ± 2.9 | 40.0 ± 2.0^†^ |
| SV (μL) | 25.9 ± 1.8 | 18.1 ± 1.3^**^ | 19.4 ± 0.4^**^ |
| EF (%) | 56.1 ± 3.8 | 35.8 ± 2.4^**^ | 49.1 ± 3.0^†^ |
| CO (mL/min) | 12.1 ± 0.7 | 7.9 ± 0.6^**^ | 9.4 ± 0.5^*^ |
| HR (bpm) | 468.9 ± 12.3 | 439.2 ± 21.9 | 484.9 ± 16.3 |

volume;s, left ventricular systolic volume; volume;d, left ventricular diastolic volume; SV, stroke volume; EF, ejection fraction; CO, cardiac output; HR, heart rate. Data are presented as the mean ± SEM. One-way ANOVA with Tukey’s test was used for statistical analysis. *p<0.05, **p<0.01 vs Sham, †p<0.05, ††p<0.01 vs ΔCM(Cys) I/R.
